# Exploring the biology of metastatic hormone-sensitive prostate cancer: on the road to precision medicine

**DOI:** 10.1172/JCI200920

**Published:** 2026-02-02

**Authors:** Alice Bernard-Tessier, Himisha Beltran

**Affiliations:** 1 Department of Medical Oncology, Dana-Farber Cancer Institute, Boston, Massachusetts, USA.; 2 Gustave Roussy, Département de médecine oncologique, Villejuif, France.

## Abstract

Metastatic hormone-sensitive prostate cancer (mHSPC) is a clinically and molecularly heterogeneous disease. Recent insights into the biology underlying disease presentation, volume of disease, and response to therapies are starting to point toward biomarkers to improve selection for intensified and deintensified treatment strategies. In addition, the therapeutic landscape is rapidly changing, with new biomarker-driven studies targeting genotype (e.g., *BRCA* or *PTEN* mutant) and phenotype (e.g., prostate-specific membrane antigen status) in development for mHSPC. A better understanding of tumor heterogeneity, clonal evolution, and metastatic homing in prostate cancer will hopefully inform future strategies for local and systemic disease control, personalized monitoring strategies, and improved patient outcomes.

## Introduction

Prostate cancer is the most common malignancy among men. Although its overall lethality is mitigated in part by effective screening programs and its unique biological characteristics, including an often indolent progression particularly in elderly men with competing causes of death, prostate cancer still remains a leading cause of mortality worldwide (1). Furthermore, there is a projected global increase in both the incidence and mortality from prostate cancer in the coming years (2). In the United States, there are an estimated 35,770 deaths from prostate cancer per year (3). Of particular concern is the finding that the incidence of metastatic and incurable disease at prostate cancer diagnosis is rising (4). Currently, among men who ultimately die from prostate cancer, approximately half are initially diagnosed with de novo metastatic hormone-sensitive prostate cancer (mHSPC), despite this disease presentation accounting for only 4%–20% of all prostate cancer diagnoses (5–7). Of those diagnosed with high-risk clinically localized prostate cancer and treated with local therapy, up to 30%–50% ultimately relapse with mHSPC (8, 9).

mHSPC is a clinically heterogeneous disease, with consistently observed differences in outcome in clinical trials based on disease presentation (i.e., metachronous vs. synchronous metastases) and volume of disease (i.e., low vs. high volume) (10). For instance, the median overall survival for patients with metachronous, low-volume disease is approximately 8 years, whereas median survival for de novo high-volume disease is only 3–4 years (11, 12). The most common definition of “high volume” in clinical trials of mHSPC is the presence of at least 4 bone metastases (with >1 outside the spine or pelvis) and/or visceral metastases (13). Treatment strategies are often tailored based on these prognostic clinical characteristics. The success of doublet therapy with androgen deprivation therapy (ADT) and androgen receptor (AR) pathway inhibitor (ARPI) therapy has been clearly demonstrated for all patients with mHSPC independent of disease presentation or volume status (14), but further intensification with the addition of docetaxel chemotherapy (i.e., triplet therapy) is often reserved for those with de novo high-volume mHSPC (11, 12). On the contrary, some patients with low metastatic burden who relapse years after local therapy do quite well overall and may even benefit from treatment holidays, a focus of ongoing clinical trials. The mHSPC therapy clinical trial landscape is rapidly evolving and also includes trials that evaluate newer biomarker-driven strategies, adding PARP inhibition to standard ADT plus ARPI therapy for those with *BRCA* gene mutations, adding AKT inhibition for *PTEN* loss, and adding prostate-specific membrane antigen–directed (PSMA-directed) radioligand therapy based on PET imaging selection.

Concurrently, advances in PET imaging have been redefining the distinction between metastatic and nonmetastatic disease as well as oligometastatic and polymetastatic disease states (15). In recent studies, stage migration with reclassification of disease from low volume to high volume occurred in 13%–22% of cases when using PSMA-PET criteria (16, 17). Notably, one study observed that 30% of cases defined by conventional imaging as low-volume disease were downstaged as nonmetastatic disease by PSMA-PET scan, raising the question of whether the current standard of using conventional imaging is accurate for staging (17). In addition, a distinct clinical entity has emerged with oligometastatic disease seen on PSMA-PET scan but not by conventional imaging. This evidence raises a critical question of whether we should consider mHSPC as a binary state or as a continuum.

A better understanding of the biology of mHSPC could lead to improved biomarkers for treatment selection and inform the development of novel targeted therapies. Most studies investigating prostate cancer biology have focused either on clinically localized hormone-naive disease or metastatic castration-resistant prostate cancer (mCRPC). Here, we explore the emerging biology underlying mHSPC and highlight current knowledge gaps.

## The kaleidoscope of metastatic prostate cancer

A kaleidoscope is an optical device that uses internal mirrors to reflect glass and create continuously changing patterns. Similarly, metastatic prostate cancer is a continuously changing and dynamic process of evolution, with its initial origins from a primary prostate tumor, heterogeneous patterns of spread, and subclonal evolution that is further shaped by therapeutic pressure. Understanding the complexity, variability, and ever-changing characteristics of metastatic prostate cancer can been challenging due to often limited evaluable tumor fraction in circulating tumor DNA (ctDNA) for sequential analysis. Nonetheless, recent systematic studies exploring primary and metastatic tissue have started to shed light into the molecular underpinnings of mHSPC.

## The genomic landscape of mHSPC

Prostate cancer is an androgen-driven disease. Prostate cancer risk is increased by aging, family history, and germline mutations involving DNA repair genes (18, 19). Dietary, lifestyle-related, and other environmental factors that induce chronic inflammation and enhanced steroid production may further contribute to risk (20, 21). Early prostate cancer genomic alterations (22) include the *TMPRSS2-ERG* gene fusion and *SPOP* and *FOXA1* mutations, which contribute to activated AR signaling important for disease pathogenesis. DNA repair gene (e.g., somatic *BRCA2* or *ATM* mutations) and tumor suppressor gene alterations (e.g., *PTEN* deletion, *TP53* mutation) can also occur early in primary tumors and contribute to tumor aggressiveness. These biological processes shaping early prostate cancer are reflected in the genomic characteristics observed in across stages.

**Figure 1:**
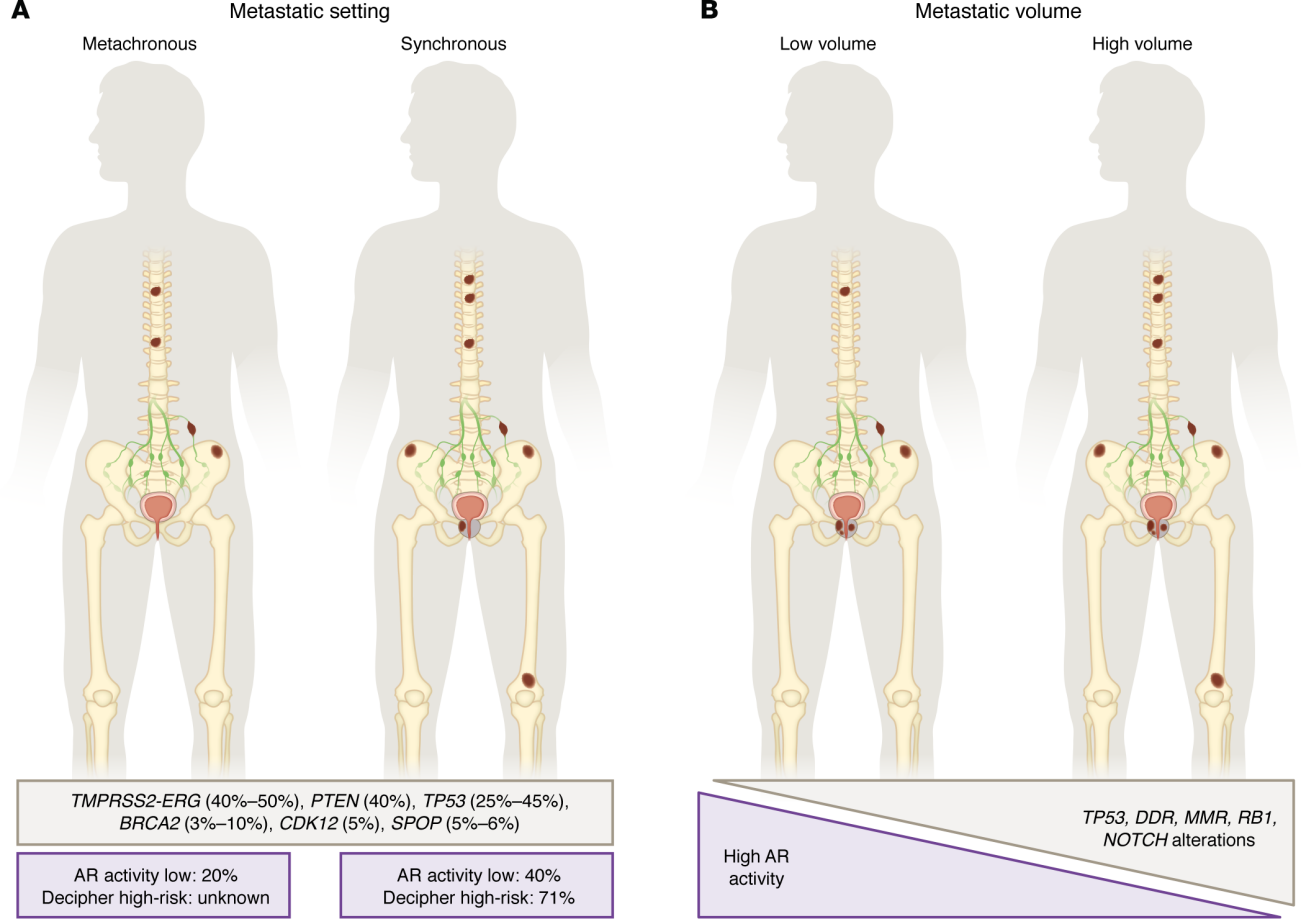
Clinical configurations of mHSPC and known associated biological features. (**A**) Synchronous versus metachronous presentation. (**B**) Low- versus high-metastatic volume. The percentage of patients with lower AR activity is shown. Decipher high-risk denotes the proportion of cases with high genomic risk scores; Decipher low-risk denotes the proportion cases with low genomic risk scores. AR, androgen receptor.

Recent studies evaluating the genomic profile of mHSPC have utilized tissues from the primary tumor and/or metastatic lesion prior to the initiation of systemic therapy for metastatic disease. In the case of de novo metastatic disease, these samples were from the time of diagnosis. For metachronous disease, primary tumor biopsy or prostatectomy specimens may have been collected several years prior to mHSPC presentation. Overall, the genomic landscape and spectrum of alterations seen in mHSPC are similar to those observed in primary prostate cancer and mCRPC, including alterations in *TMPRSS2-ERG* (40%–50%), *PTEN* (40%), *TP53* (25%–45%), *BRCA2* (3%–10%), *CDK12* (5%), and *SPOP* (5%–6%) (23–26) (Figure 1A). Homozygous loss of *RB1* is uncommon in mHSPC but heterozygous loss is reported in up to 30% of patients (27, 28). The overall frequency of higher risk alterations, including loss of tumor suppressors, is more common in mHSPC than localized disease (29, 30). As expected, *AR* gene alterations are not seen in mHSPC or localized prostate cancer without a history of prior ADT exposure. An analysis of tumors from the STAMPEDE trial of high-risk nonmetastatic and metastatic prostate cancer revealed an accumulation of somatic copy number alterations (SCNAs) correlated with the presence of metastases and associated with inferior overall survival. Specific segments, including those involving *CHD1* or *MYC*, were associated with high SCNA burden, suggesting an order in the accumulation of these events that may drive downstream SCNAs and a cell survival advantage (31).

Reports indicate an enrichment of tumor suppressor gene losses, DNA repair gene alterations, and WNT pathway alterations in patients with high versus low volume of disease (23, 26, 32) and a trend toward enrichment in the NOTCH pathway (25), cell-cycle pathway genes (25, 32, 33), and chromatin remodeling or DNA mismatch repair (23) gene alterations in high-volume disease (Figure 1B). These alterations, especially tumor suppressor gene aberrations, have been associated with clinical aggressiveness and poor prognosis (28). Enrichment in high-volume disease could potentially underly the rapid progression and relatively short duration of response to hormonal therapies in some patients. Notably, in a recent study evaluating patients with *BRCA* alterations, clinical outcomes did not differ markedly between those with low and high metastatic volume, suggesting that the prognostic weight attributed to disease volume should be balanced in conjunction with other prognostic features such as *BRCA* status (24).

Testing for germline and somatic genomic alterations in mHSPC has potential clinical implications. There are important family implications for germline testing for all patients with mHSPC, and this has been universally supported by clinical guidelines (34–36). For patients eligible for currently approved biomarker-driven therapies in mCRPC (e.g., PARP inhibition), knowing their mutation status before progression to mCRPC could aid the timely introduction of these therapies. In addition, these biomarkers could point to novel targeted strategies and could guide clinical trials in mHSPC. The recently reported AMPLITUDE trial showed that in patients with mHSPC with *BRCA* or other homologous recombination gene mutations, the combination of the PARP inhibitor niraparib and the ARPI abiraterone improved radiological progression-free survival compared with placebo and abiraterone (hazard ratio 0.63) (37). In an exploratory analysis of 323 patients with BRCA2 mutation, the hazard ratio for radiological progression-free survival was 0.46 (95% CI: 0.32, 0.66) (37). Based on these results, niraparib plus abiraterone was approved by the FDA in December 2025 for patients with BRCA2-mutated mHSPC. The CAPITELLO-281 phase III trial evaluated the addition of the AKT inhibitor capivasertib to abiraterone and ADT in patients with mHSPC harboring PTEN loss defined by the presence of ≥90% malignant cells that lacked staining by immunohistochemistry (38).Approximately 25% of screened patients (1,519 of 6,003 patients) had PTEN deficiency (39). There was a statistically significant improvement in radiological progression-free survival with the addition of capivasertib (hazard ratio 0.81), with greater benefit observed in patients with higher levels of PTEN loss (e.g., >95%, >99%, >100%). As also seen in mCRPC (39), these data confirm not only the importance of this biomarker, but also the potential need to refine detection methods beyond a simple positive/negative assessment. Moreover, prostate-specific antigen (PSA) progression events were less frequent than clinical progression in both arms, highlighting a distinct pattern of disease evolution in PTEN loss tumors that may help inform the optimal type of surveillance (38).

Although relatively uncommon, the presence of *SPOP* mutations in mHSPC has been associated with more favorable outcomes in patients receiving ADT plus ARPI but not with patients receiving ADT plus docetaxel, suggesting a potential predictive value for response to ARPI (40). This finding aligns with preclinical studies indicating hormone sensitivity linked to *SPOP* status (41). *SPOP* is a E3-ubiquitin ligase substrate adaptor that mediates the protein stability of AR and its transcriptional coactivators. However, it has not yet been demonstrated whether the presence of *SPOP* mutations allows omission of docetaxel when combined with ARPI or how *SPOP* status could help inform other future deintensification treatment strategies.

**Figure 2:**
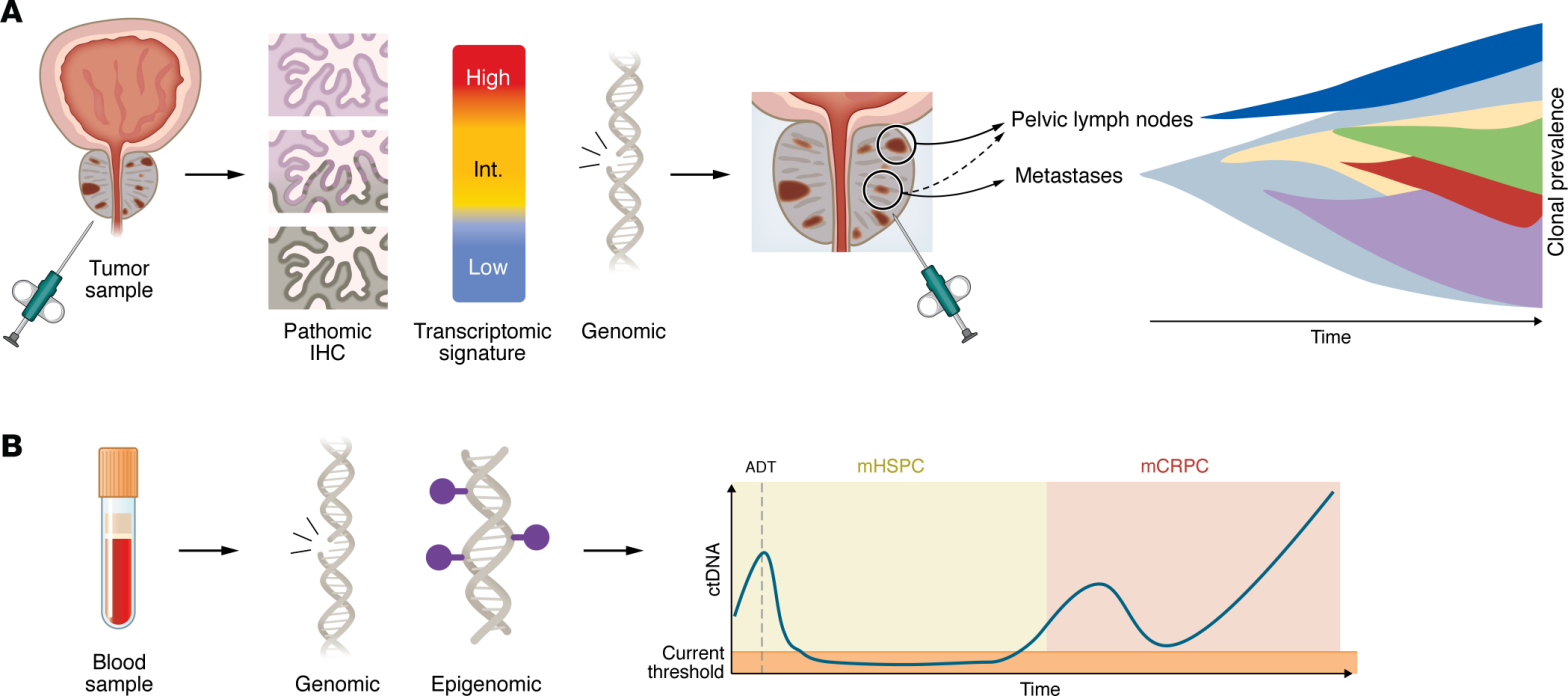
Approaches used to capture tumor biology and their limitations. (**A**) Tissue samples provide detailed molecular information but fail to reflect clonal evolution over time. (**B**) At the time of diagnosis of hormone-sensitive cancer, the ctDNA fraction is often low and does not allow the same depth of analysis as tissue samples. ADT, androgen deprivation therapy; ctDNA, circulating tumor DNA.

The best method to test for somatic genomic aberrations in mHSPC is not well defined. Metastatic tumor biopsies are often challenging to perform, especially for patients with low burden of metastatic disease or sclerotic bone metastases. Primary tumors are often used, since actionable aberrations such as *BRCA* loss or mismatch repair deficiency are typically early clonal drivers and present in the primary (22) (Figure 2). Nonetheless, it is possible that the dominant lesion is not evaluable and/or that acquired alterations that occur with metastatic spread are not captured. ctDNA analysis is more challenging in mHSPC compared with mCRPC, especially in lower tumor burden states, and ctDNA abundance rapidly declines after initiation of systemic hormonal therapy (26), limiting the feasibility of comprehensive or sequential ctDNA analysis.

## Tracking the clonal origin of metastatic prostate cancer

It has been suggested by prior studies, including autopsy studies, that most prostate cancer metastases have a monoclonal origin traceable back to the primary tumor (42–46). In other words, just one clone within the primary prostate, even in the case of multifocal and multiclonal disease, is responsible for lethal disease. This clone may acquire additional alterations with tumor evolution and treatment resistance (47). Metastasis-to-metastasis seeding further contributes to intrapatient tumor heterogeneity seen in advanced CRPC (42).

One challenge in identifying the clone within the primary tumor with metastatic and lethal potential lies in the fact that the prostate may harbor multifocal and spatially, morphologically, and genomically distinct tumor foci (48). In a recent study of 43 patients who underwent prostatectomy following an mHSPC diagnosis, most of the primary tumor populations were not detected in the corresponding metastatic samples (49). However, despite this challenge of multiclonal heterogeneous prostate tumors, it is likely that most major oncogenic and currently targetable drivers, found in prostate tumor samples and in serial ctDNA, are clonal and present in the majority of metastases (50) and retained during disease progression (26, 29, 49).

One could hypothesize that distinct primary events or evolutionary trajectories shape the clinical presentation of mHSPC, whether de novo or metachronous. These trajectories may follow a linear progression in which later events within the primary clone grant the capacity to spread or a divergent evolutionary pattern where early events drive dissemination through multiple waves (51). In a phylogenic study evaluating patients with mHSPC, the origin of regional lymph node metastases could be evolutionarily traced back to extraprostatic tumor cells, whereas bone metastases arose from both extra- or intraprostatic cells at both early and late evolutionary stages (52). There may also be continued seeding of metastatic sites in waves from an intact primary tumor, providing biologic rationale for treating the primary tumor in patients with metastatic disease. This might be most relevant in patients with limited metastatic burden, before tumors have had a chance to evolve and be dominated by more aggressive disease clones. Indeed, the STAMPEDE and PEACE-1 studies have demonstrated a survival advantage for treating the primary tumor with radiotherapy in patients with low-volume mHSPC (53, 54).

It still remains to be fully understood whether mHSPC lesions are dependent on driver events in the original clones released from the primary or if they are dependent on acquired subclonal alterations or even derived from another metastasis (55). It is likely that complex, multifaceted mechanisms contribute to the establishment of the metastatic niche and continued tumor growth and spread.

## Biology of metastasis

**Figure 3:**
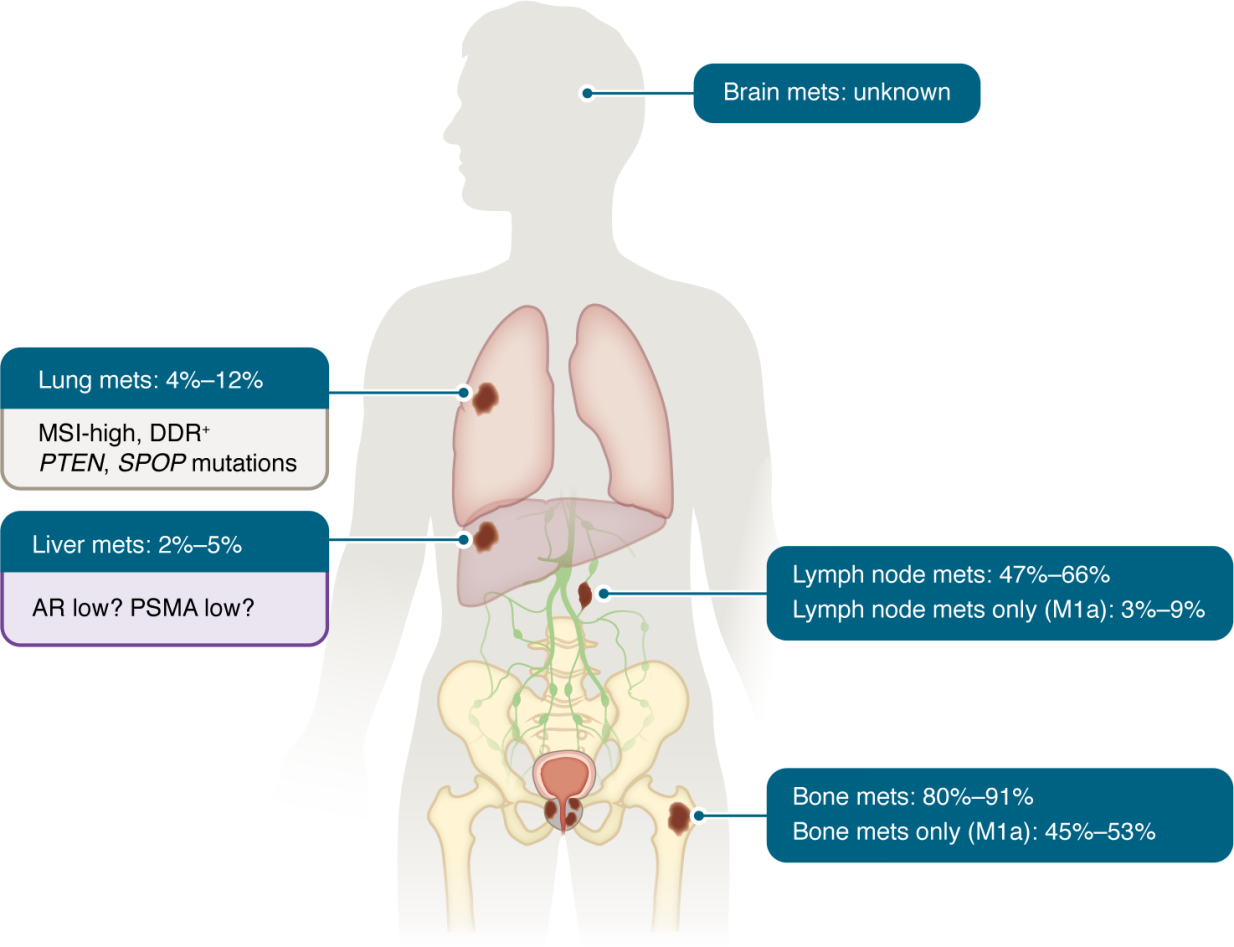
Predominant sites and characteristics of mHSPC metastases. See refs. 110–112.

The prognosis of mHSPC is linked with not only with the volume of disease, but also with the anatomic sites of metastasis. For instance, patients with lymph node–only or low-volume lung-only disease typically have a more favorable outcome compared with those with extensive bony disease or liver metastases (56). The mechanisms underlying metastatic homing to distinct sites likely rely both on tumor-intrinsic features and the tumor microenvironment, yet these molecular mechanisms have not fully been characterized in the context of mHSPC (Figure 3).

The most common sites of metastasis in prostate cancer are the bone and lymph node (56). Although no direct one-to-one comparisons have been performed and most available data derive from pelvic lymph node analyses, current evidence suggests that the genomic landscapes of prostate cancer metastases limited to lymph nodes or bone are largely comparable, with overlapping key genetic alterations across these most common metastatic sites (52, 57, 58). Patients with lymph node–only metastasis have a more favorable prognosis, which may be related in part to clonal evolution, with nonindex primary clones metastasizing to lymph nodes but not responsible for lethal disease (52, 57). Depending on the extent, low-volume lymph node metastases are also more amenable to treatment with nodal dissection or pelvic radiation with hormonal therapy (59).

Bone metastases arise through hematogenous spread. The bone microenvironment creates a niche that can support tumor dormancy and eventual outgrowth. Factors, such as TGF-β, IGF, and others, support bone remodeling and changes in both osteoblastic and osteoclastic activity, resulting in mostly sclerotic bone lesions (60). Lytic lesions can also occur but are less common (61). The α emitter Radium-223, which targets areas of bone turnover, is approved for the treatment of patients with CRPC and bone metastasis and is currently under investigation in mHSPC (NCT04037358, NCT03304418).

The biology underlying low-volume, lung-only prostate cancer metastasis, and why this tends to associate with more favorable prognosis, is not fully known. One retrospective analysis of lung-only metastases identified enrichment of mismatch repair and other DNA repair mutations (62), though this was not confirmed in another cohort that showed genomic profiles similar to those of localized tumors with common *PTEN* and *SPOP* alterations (63). However, both cohorts demonstrated prolonged survival compared with historical control cohorts, underscoring the importance of distinguishing these cases from other visceral metastases. Indeed, due to their small numbers, visceral metastases are often grouped together in phase III trials, limiting insights into the biological drivers and treatment effects by specific site. A post hoc analysis of the LATITUDE phase III trials comparing ADT versus ADT plus abiraterone in high-risk mHSPC patients showed that the addition of abiraterone improved progression-free survival in patients with lung metastases but not those with liver metastases (64). In contrast, triplet therapy combining ADT with ARPI and docetaxel might offer benefit for patients with liver metastasis (12, 65).

While liver metastases are not common in mHPSC, their presence is negatively prognostic, as also observed in mCRPC (66, 67). In CRPC, the presence of liver metastasis has been linked with AR independence, relatively lower AR expression levels, and small cell neuroendocrine features (68, 69). In mHSPC, less is known about the biology of liver metastasis and whether there is biologic overlap with those seen in mCRPC. A recent analysis of 7,082 prostate cancer specimens constituting primary prostate and distant metastasis showed a lower *FOLH1* (coding for PSMA protein) expression in liver as well as lung metastases compared with lymph node metastases and a positive correlation of *FOLH1* gene expression with AR signaling scores (70). Despite the heterogeneity of the population, these results endorse the latter hypothesis. Brain metastasis are very rare in prostate cancer but may increasingly be identified due to better imaging modalities (e.g., PET) and, in the CRPC setting, the systemic therapies may improve the control of extracranial disease progression. (71). In one study, whole-genome sequencing revealed a high diversity of complex structural alterations, chromothripis, and chromoplexy events in prostate cancer intracranial metastases (72).

The tumor microenvironment of mHSPC differs from that of mCRPC, reflecting distinct immune compositions and functional states (73, 74). A comparative analysis of a small cohort of mHSPC patients has shown that while bone, lymph node, and liver metastases display similar degrees of immune infiltration, lung metastases appear relatively immune-depleted, suggesting site-specific immune dynamics (75). Such variations may in part be shaped by tumor-intrinsic genomic alterations that influence immune signaling pathways and microenvironmental interactions (76, 77). Despite immune infiltration, all randomized studies testing the addition of immune-checkpoint inhibitors to ARPI or docetaxel have failed to demonstrate a survival improvement (78–80). Considering the modulatory effects of AR signaling on immune cells (75, 81), together with the pivotal role of macrophages and exhausted T cells in shaping immune responses in mCRPC (82, 83), it is critical to better characterize the mHSPC immune landscape when rationally designing combination strategies targeting both tumor cells and the immune microenvironment, particularly in the context of T cell engager therapies.

Overall, additional studies are needed to understand how tumor biology contributes to the sites of prostate cancer metastases in mHSPC, the impact of the microenvironment, and how these factors affect the response to systemic therapies and outcomes.

## Oligometastatic disease

Oligometastatic disease is thought to represent an intermediary state between the primary localized cancer and widespread metastasis (84). In prostate cancer, oligometastatic disease is traditionally defined by counting the number of metastatic lesions on conventional scans (CT and bone scan), with varied definitions of cutoffs in clinical trials ranging from 3 to 5 metastases (85). This definition is now evolving with the increased use of more sensitive PSMA-PET imaging for staging.

The clinical and biologic spectrum of oligometastasis is also heterogenous. There are some cases, particularly patients with metachronous oligometastatic disease detected on PSMA-PET long after treatment of their primary, where there is a more indolent course. The oligometastatic sites in these scenarios might have lower metastatic potential, and treatment of these sites by metastasis-directed therapy (MTD) may provide a relatively durable disease control. In other cases, detection of oligometastasis is just the tip of the iceberg, with more aggressive disease ready to dominate and widespread metastasis developing soon after. It is challenging to track the natural history of oligometastatic disease, especially since therapy is not standardized. Furthermore, as our ability to detect metastatic lesions improves with PET imaging, it remains unclear whether all patients with oligometastatic disease detectable only by PET-PSMA require the same systemic therapy regimens as those given for low-volume mHSPC. The development of biomarkers could potentially help identify which patients benefit the most from MTD and could guide the type and duration of concurrent systemic therapy.

Prior studies have suggested that the number and type of genomic aberrations differ in de novo versus metachronous oligometastatic disease as well as based on the number of metastases. The presence of *TP53* mutation and DNA repair gene alterations in primary tumors, for instance, has been associated with a greater number of metastatic lesions (32), and similar mutational profiles were seen in polymetastatic and de novo mHSPC with comparable time to CRPC, suggesting that these entities may share similar disease biology. Progression patterns after oligometastasis treatment can also be affected by genomics. In a recent study, *TP53* mutation and *RB1* mutation were associated with a higher rates of polyprogression, and bone failure was more common in cases with *TP53* mutations and less common in cases with *SPOP* mutations (86).

As key early drivers, such as *CHD1* loss (87), *MYC* amplification (88), *PTEN* loss (89), and *BRCA* mutations (90), are known to be individually associated with prostate cancer aggressiveness in localized disease, it is plausible that their presence may also predict metastasis patterns and outcomes. The integration of these genomic markers represents a promising approach for earlier detection and improved risk stratification. Future studies that extend beyond genomics to incorporate gene expression, proteomics, and epigenetics may also provide additional insights.

A better understanding of tumor biology could help answer critical questions about the treatment of oligometastatic prostate cancer. These include (a) who benefits most from MDT; (b) what is the optimal systemic therapy type and duration; (c) in whom could we delay systemic therapy (ADT-free survival); (d) are there patients with metastases beyond bone or lymph node who would benefit from MTD (e.g., solitary liver metastasis); and (e) how many times should we treat (for patients who have recurrent oligometastasis)?

## Phenotypic variants

In addition to genomic subtypes of prostate cancer, phenotypic variants can also be observed, including aggressive histologic variants associated with cribriform/intraductal patterns in localized disease and neuroendocrine prostate cancer (NEPC) features in mCRPC. Pure small cell carcinoma of the prostate rarely arises de novo and is more commonly observed in later stages of prostate cancer progression as a mechanism of treatment resistance. While tumors can be heterogenous, metastatic tumor biopsy and autopsy studies suggest that up to 15% of late-stage mCRPC tumors harbor NEPC features (91, 92). NEPC is associated with morphologic characteristics of small cell carcinoma, expression of classical neuroendocrine markers such as INSM1 and synaptophysin, and often low or absent AR expression (93). NEPC is also enriched with tumor suppressor losses especially *RB1* and *TP53* and has distinct transcriptomic and epigenetic features (94). Notably, transcriptomic features of NEPC have been observed, even at prostate cancer diagnosis, especially in high-grade localized tumors with low PSA levels (95). Furthermore, mixed adenocarcinoma-NEPC features are sometimes seen in localized or metastatic HSPC. Whether these features predict future histologic transformation to NEPC in the mCRPC setting has not been established. In the PEACE-1 trial, 26% of de novo mHSPC tumors had neuroendocrine histologic features and/or expressed classical neuroendocrine markers by immunohistochemistry, while 1.4% and 0.8% of patients had pure NEPC or double-negative tumors (defined as adenocarcinoma lacking both AR and NEPC markers), respectively. Baseline neuroendocrine marker expression or the presence of at least two genomic alterations involving *TP53*, *RB1*, and *PTEN* were associated with worse outcomes, although none of these biomarkers could predict abiraterone survival benefit (96). Mirroring tumor suppressor gene losses and NEPC features, a high level of Ki-67 immunopositivity, indicating proliferation, has also been correlated with worse outcomes (97).

While NEPC features, histologically or transcriptionally, suggest more aggressive disease and less AR dependence, treatment of these cancers in mHSPC currently follows mHSPC guidelines, especially since AR, PSA, and PSMA are often still expressed. We can hypothesize that mHSPC tumors with pathologic or molecular overlap with castration-resistant NEPC may represent early aggressive clones that are selected for in the context of therapy and/or are primed to develop lineage plasticity and future transformation to pure NEPC. Further studies are needed to establish biomarkers associated with NEPC in the mHSPC setting and whether triplet therapy or even quadruplet therapy (e.g., ADT, ARPI, docetaxel, carboplatin) should be given. We would consider closer monitoring of such patients with imaging (not relying only on PSA) and a low threshold to rebiopsy at progression to look for a dominant NEPC pattern at the time of mCRPC.

## Transcriptomic features

Prostate cancer phenotypes can also be distinguished based on transcriptomic features, including expression of gene signatures or certain targets. In mHSPC, the AR transcriptional program can differ according to metastatic site and may influence treatment efficacy and resistance mechanisms. mRNA profiling of primary tumors from patients enrolled in the CHAARTED trial (98) showed that de novo metastatic cases display a much higher frequency of AR-low tumors (around 40%) compared with localized disease, where AR-low tumors account for only about 10% of cases (99). A retrospective study further highlighted heterogeneity within metastatic disease (100). Tumors in individuals diagnosed with synchronous mHSPC had lower AR transcriptional activity and a reduced hallmark androgen response signature. Interestingly, this biology appeared more closely linked with tumor volume than clinical timing, since differences were mainly evident in low-volume disease, while high-volume presentations did not show clear separation in AR signaling profiles. Overall, studies have suggested the AR signature is prognostic, and high AR activity has been associated with better outcomes compared with lower activity in metastatic and nonmetastatic disease (97).

PSMA is considered an indirect target associated with AR signaling (68). Analysis of mRNA expression in a large cohort of 5684 hormone-naive prostate tumors supported an association between PSMA (encoded by the *FOLH1* gene) expression patterns in primary disease and clinical and biological features (101). Patients with PSMA-low tumors had overall lower AR activity and a more basal phenotype. Conversely, higher PSMA expression was associated with greater AR activity and, clinically, with improved responses to ADT in the setting of biochemical recurrence, resulting in longer cancer-specific survival compared with those with PSMA-low disease. Notably, low PSMA tumors were also associated with reduced response to hormonal therapy and potentially increased benefit for intensification with docetaxel (101). Lower and more heterogenous PSMA expression has also been observed in liver metastases, in part due to epigenetic suppression (70, 102). It is possible that higher PSMA expression (in tumors or by imaging) in patients with mHSPC may be associated with improved response to PSMA-directed radioligand therapy such as Lu-PSMA-617. The phase III PSMAddition trial evaluating ADT plus ARPI with or without Lu-PSMA-617 met its primary endpoint, showing a 28% reduction in the risk of radiographic progression or death with the addition of Lu-PSMA for patients with PSMA-PET–positive mHSPC (103).

The PAM50 gene signature classifies tumors into luminal and basal subtypes and has been studied in both breast and prostate cancers (104). In the CHAARTED trial of patients with mHSPC treated with ADT with or without docetaxel, luminal B (50%) and basal (48%) subtypes were predominant; patients with luminal B tumors seemed to benefit more from the addition of docetaxel compared with those with basal subtype (98). Low AR activity was also negatively prognostic in this study.

The Decipher test, a 22-gene expression classifier that is prognostic in clinically localized prostate cancer (105), has also been studied in mHSPC with an overall higher proportion of high genomic risk scores seen in metastatic cases compared with localized disease, 71% and 16.5%, respectively (98). In the STAMPEDE trial, Decipher score was higher in tumors with lymph node involvement (M0N1) and in metastatic disease (M1) compared with localized disease (M0N0), with a similar distribution across tumor volume (106). Moreover, Decipher score was strongly associated with overall survival (97), independent of tumor volume. Gene expression signatures associated with plasticity, immune activity, and tumor plasticity were also prognostic (97).

In the STAMPEDE trial, although Decipher score was prognostic, benefit was seen for the addition of abiraterone to ADT in both Decipher-high and -low cases. With docetaxel, a high Decipher score identified patients more likely to benefit from docetaxel, with a biomarker treatment interaction effect. PTEN inactivity by transcriptome analysis was also associated with increased sensitivity to docetaxel, without overlap with the Decipher classifier (97). These results highlight the potential of molecular biomarkers to guide clinical decision-making and potentially optimize therapeutic strategies individualized to tumor biology.

In the future, spatial transcriptomics might provide complementary insights to better capture tumor phenotype and heterogeneity, with simultaneous gene expression and localization also enabling higher resolution insights into the complex interplay between tumor cell populations, stroma, and immune infiltrates regionally within tumors (107). This approach has the potential to unravel the influence of the metastatic niche on the transcriptome, including AR signaling and cell-surface antigen heterogeneity.

## How can mHSPC biology provide information about progression patterns?

One of the most accessible surrogate measures of AR transcriptional response is serum PSA, as PSA is a direct target gene of the AR. Deeper declines of PSA under androgen-targeted therapies, particularly when it achieves thresholds below 0.2 ng/mL at 6–8 months after treatment initiation, have consistently correlated with improved outcomes across multiple phase III trials, confirming PSA nadir as a prognostic factor irrespective of the chosen intensification approach: ADT plus docetaxel (108), ADT combined with ARPI (109), and triplet regimens (110). Ongoing trials, such as PEACE6 (NCT06496581) and LIBERTAS (NCT05884398) or DE-ESCALATE (NCT05974774), are assessing its role as a predictive biomarker for therapy intensification or deintensification. Beyond PSA, additional future biomarkers may help select patients for deintensification or intermittent strategies — by reducing the duration of long-term AR blockade — such as PSMA-PET response, ctDNA negativity, or, even potentially, more sensitive ctDNA platforms, such as emerging minimal residual disease assays that have not yet been extensively evaluated in the context of mHSPC. Identifying patients with suspected non-AR-driven disease based on phenotype, epigenetics, or transcriptomics could also guide personalized monitoring, including the type and frequency of imaging and ctDNA platforms beyond PSA/PSMA.

## Conclusions

The treatment landscape of metastatic prostate cancer is rapidly changing. The choice of therapy is often based on clinical features (e.g., disease presentation and volume). An increased understanding of the biology of mHSPC may help improve patient selection for existing therapies and newer biomarker-driven approaches. Collectively, the kaleidoscope of clinical and biologic presentations of prostate cancer suggests distinct disease trajectories, potentially reflecting different primary clonal events or divergent evolutionary patterns. Identifying early molecular drivers of progression and the presence of aggressive clones at diagnosis could lead to improved strategies for local control and disease monitoring. Overall, the goal of therapy in metastatic prostate cancer is for patients to live longer and live better. With improved detection, biomarkers,and treatment of mHSPC, achieving long term remission (a therapy-free window)—and even cure for select individuals—might be a tangible future goal.

## Version 1 02/02/2026

Electronic publication
